# Sex differences in association between cognitive impairment and clinical correlates in Chinese patients with first-episode drug-naïve schizophrenia

**DOI:** 10.1186/s12991-021-00347-1

**Published:** 2021-04-21

**Authors:** Na Zhao, Xiao Hong Wang, Chuan Yi Kang, Yue Zheng, Li Ying Yang, Tie Feng Guan, Yun Xia Bai, Ran Wei, Hunter C. Hinman, Xiang Yang Zhang

**Affiliations:** 1 Department of Psychiatry, The First Affiliated Hospital of Harbin Medical University, Harbin, Heilongjiang Province China; 2 Psychological Clinic, The First Hospital of Yichun, Yichun, Heilongjiang Province China; 3 Department of Clinical Psychology, Suzhou Guangji Hospital, The Affiliated Guangji Hospital of Soochow University, Suzhou, Jiangsu Province China; 4 Department of Psychiatry and Behavioral Sciences, The University of Texas Health Science Center At Houston, Houston, TX USA; 5 CAS Key Laboratory of Mental Health, Institute of Psychology, Chinese Academy of Sciences, Beijing, China; 6 Department of Psychology, University of Chinese Academy of Sciences, Beijing, China

**Keywords:** Cognition, First-episode drug-naïve patients, MCCB, Schizophrenia, Sex difference

## Abstract

**Background:**

Schizophrenia is a complex mental illness with significant sex differences. Cognitive impairment is common in patients with schizophrenia, even in remission. This study was designed to examine the sex differences in the relationship between cognitive impairment and clinical correlations with first-episode drug-naïve (FEDN) schizophrenia.

**Methods:**

93 FEDN patients (male/female = 45/48) and 160 controls (male/female = 74/86) were enrolled to compare the sex differences in cognitive functions measured by the MATRICS Consensus Cognitive Battery (MCCB). Positive and Negative Syndrome Scale (PANSS) and Hamilton Depression Scale (HAMD) were used to evaluate patients' clinical symptoms. We compared cognitive impairment with sociodemographic characteristics and measures of different genders, as well as group-by-sex interactions.

**Results:**

Our results showed that male patients had significantly lower scores for symbol coding, digital sequence, and verbal learning than female patients, while the healthy controls showed similar sex differences. In female patients, multiple linear regression analysis confirmed that PANSS negative symptoms and general psychopathology scores, HAMD total score, and education level were independent contributors to MCCB total score. In male patients, only education was an independent contributor to MCCB total score.

**Conclusions:**

These findings revealed significant sex differences in cognitive impairments and clinical symptoms in FEDN, which will be worthy of a follow-up study of schizophrenia in the future.

## Introduction

Schizophrenia is a complex neuropsychiatric disease with noticeable sex differences. There is growing evidence that sex differences are present in almost all aspects of schizophrenia, including demographics, symptoms, social functioning, and treatment responses [1]. A majority of studies have shown that women have a later age of onset, fewer negative symptoms, and better reactions to antipsychotic drugs than men with schizophrenia [2], while men show more dysfunction and cognitive impairment, more substance abuse, and antisocial behavior [3]. Many studies have suggested that bio-psycho-social differences such as genetic susceptibility and abnormalities in neurodevelopment may play an important role [4–6]. Furthermore, cognitive dysfunction still exists during the remission period of schizophrenia, indicating that the clinical treatment effect is not sufficient in this domain, which constitutes the leading health, economic and social burden [7]. Therefore, the study of cognition and sex differences in patients with schizophrenia is essential for understanding the basis of neurobiological substrates.

Multiple pieces of evidence suggest that cognitive impairment is a core feature that often occurs in the lifetime of schizophrenia [8, 9], and involves a wide range of deficits, including language, attention, memory, processing speed, and executive function [10, 11]. Cognitive dysfunction is significant because it is related to functional outcomes. Many studies support gender as a factor in controlling this correlation. Among 360 patients with first-episode psychosis, Li found a significant correlation between positive symptoms, short-term attention, and selective attention in male patients, while the correlation between memory and negative symptoms was more significant in female patients [12]. Another study showed that men generally performed poorly in verbal learning and memory, while women showed more extended responses to working memory tasks [13]. However, in a study by Ayesa-Arriola, there was no difference in neuropsychological performance between sexes during the first psychotic episode [14].

In China, there are few studies on sex differences and cognition of patients with schizophrenia. A recent study suggested that cognitive deficits are similar, and there is considerable heterogeneity between sexes in terms of symptoms and cognition [12]. In another present study, we found significant sex differences in many aspects of cognitive deficits with chronic schizophrenia [15]. Previous research from our group selected schizophrenia patients with or without diabetes and found that men performed poorly in immediate memory and delayed memory in both groups. Male patients with schizophrenia had the low supportive cognitive ability, regardless of whether they had diabetes [16]. Our study indicates that the first-episode drug-naive and chronically medicated patients with schizophrenia have cognitive dysfunction, showing that MATRICS Consensus Cognitive Battery (MCCB) is a sensitive measurement tool for measuring cognitive impairment in Chinese patients with schizophrenia. It also suggests that cognitive impairments exist in the early stage of schizophrenia [17], some of which may be more severe in the stage of chronic disease [18].

Currently, the research results are inconsistent, and the pathophysiological mechanisms at play are still not exact. These studies' shared and contradictory findings show the sex differences in cognitive impairment of patients with first-episode drug-naïve schizophrenia worthy of further research. There are many explanations for these differences related to genetic susceptibility and neurodevelopment, or bio-psycho-social factors [19–22]. Besides, culture may also play an essential role in sex differences in schizophrenia [2, 23, 24]. Medication may also affect the impact of cognitive function on the treatment outcome [6]. These differences can be better observed by excluding drug interventions in patients with first-episode drug-naïve schizophrenia.

To the best of our knowledge, there are few studies on the sex differences in cognitive impairment in first-episode drug-naïve schizophrenia. Therefore, the purpose of this study is to explore: (1) whether Chinese patients with FEDN schizophrenia had cognitive impairment compared to healthy controls; (2) whether cognitive impairment in schizophrenia showed sex differences; and (3) whether the sex differences in cognitive impairment is significantly correlated with clinical symptoms or general characteristics.

## Methods

### Participants and study setting

A total of 389 subjects were enrolled in this study, including 200 FEDN schizophrenia patients and 189 normal controls. And then, 107 patients and 29 controls were eventually excluded due to incomplete data (*N*_controls_ = 12), incomplete assessment of PANSS (*N*_patients_ = 30), and incomplete cognitive assessment of MCCB (*N*_patients_ = 78, *N*_controls_ = 17); there was one patient who missed both PANSS and MCCB assessment. Finally, 93 schizophrenia patients and 160 normal controls were included in the analysis. The study was approved by the Institutional Review Board of Beijing HuiLongGuan Hospital. The informed consent form was written before their inclusion.

According to the Diagnostic and Statistical Manual of Mental Disorders (DSM-IV), the sample included patients ranging from 16 to 60 years old who met the diagnosis of schizophrenia according to the Diagnostic and Statistical Manual of Mental Disorders (DSM-IV). Two independent psychiatrists diagnosed each patient. All patients were first-time. They also met the following criteria: Han nationality, the duration of symptoms is less than 60 months, and no antipsychotic drugs were taken before this treatment. Individuals with other mental illnesses were excluded from this study.

The subjects of the control group came from the local community in Beijing. The interview was used to assess the status of the subjects to meet the requirements of this study. None of them had a family history of psychotic disorder. All the control cases were Han nationality, and 160 normal controls were recruited from nearby during the same period, including 74 males and 86 females.

### Measures

The subjects were evaluated by a detailed questionnaire, including general condition, medical history, sociodemographic characteristics, and treatment stage. The Hamilton Depression Scale (HAMD) was used to evaluate depressive symptoms, and the Clinical Global Impression (CGI) was an overall assessment scale.

Positive and negative symptoms were assessed by the Positive and Negative Syndrome Scale (PANSS), conducted by two psychologists with more than 5 years of working experience. The psychologists administering PANSS were blinded to the control versus schizophrenia group status of the subjects. After that, the intermediate raters' correlation coefficient is kept above 0.8 in the repeated evaluation of PANSS throughout the research. Three subscale models were proposed, including positive symptom subscale (P), negative symptom subscale (N), and general psychopathology subscale (G).

MATRICS Consensus Cognitive Battery (MCCB) is approved by the FDA to evaluate cognitive deficiencies and is a feasible endpoint indicator for clinical trials [25]. MCCB selected ten sub-tests from more than 90 tests, representing seven cognitive domains. It includes six factors extracted from the multi-factor analysis of schizophrenic cognitive operations: Speed of Processing, Attention, Working Memory, Verbal Learning, Visual Learning, Reasoning, and Problem-solving. The seventh cognitive domain is Social Cognition, which is a neurocognitive intermediary that reflects functional outcomes. These tests have high test–retest reliability, and most of them are above 0.70. In 2008, Professor Yu Xin introduced MCCB into China and conducted normative research to adjust to Chinese populations. In 2012, he began to write a specification manual, and in 2014 the MCCB China Model Manual was published. The standardized T score for each subject is calculated, which accounts for inconsistency in translation and makes the MCCB an appropriate measure in China [26].

### Statistical analysis

Demographic and clinical data were compared using variance (continuous variables) and Chi-square test (categorical variables). The term “Group” refers to the categorization of FEDN schizophrenia versus controls, and “Sex” refers to men versus women with FEDN schizophrenia and men versus women controls. When significance was found in ANOVA, the effect of age, education, smoking, and marital status was tested between the FEDN schizophrenia and the controls. To adjust the influence of these variables on cognition, analysis of covariance (ANCOVA) was further assessed between groups. For the cognitive comparisons, we compared the MCCB total score and the effects of ten separate domains on group and sex, as well as group-by-sex interactions on each item. Associations between demographic, clinical characteristics, BMI, and MCCB total score and ten index scores were assessed by Pearson correlation coefficients in male and female patients separately. We compared the total score of MCCB with sociodemographic characteristics and measures of different genders. We used the Bonferroni corrections to adjust for multiple testing. Stepwise multivariate analysis using MCCB total score as the dependent variable was used to investigate the impact of a range of variables. Through the research of related factors, several influencing factors were identified. Seven items of education, BMI, HAMD total score, PANSS total score, N, G, P entered the model. For sex, N and G were in the model because they strongly correlate with PANSS total score. When forming multiple collinearities, the PANSS total score was not included in the equation. The other 6 items for both male and female groups were included in the multiple linear regression model. The statistical software package for statistical calculations was the Statistical Program for Social Sciences (SPSS, version 24.0). The statistical test was considered with a two-tailed test, and the significance was set at 0.05 level.

## Results

### Demographic and clinical data

A total of 93 cases of FEDN schizophrenia and 160 cases of normal controls were included in this study. The normal control group's age was older than that of the FEDN schizophrenia group (43.54 ± 12.01 versus 26.41 ± 8.01, *p* < 0.001). The variables were comparable between the groups (all *p* > 0.05). There were 45 males and 48 females in the FEDN schizophrenia group and 74 males and 86 females in the normal control group. There was no significant difference in gender distribution (*p* = 0.743).

Table 1 shows that male patients scored higher than female patients on PANSS total score, PANSS negative symptom and general psychopathology subscale scores, and HAMD total score (all *p* < 0.05). However, the significant differences in smoking, PANSS negative symptom subscore, and HAMD total score did not pass the Bonferroni correction (Bonferroni corrected *p* < 0.05/8 = 0.00625). Smoking displayed gender differences in both the control and schizophrenia groups. Thus, we controlled for smoking in the following analyses.

**Table 1:** Demographic and clinical characteristic in FEDN schizophrenia patients by sex

	Male patients (*n* = 45)	Female patients (*n* = 48)	*F* or *X*^2^	*p* value
Age (years)	25.47 ± 8.57	27.29 ± 7.43	1.209	0.274
Education (years)	12.49 ± 3.25	12.94 ± 3.48	0.411	0.523
Nonsmoker/smoker	35/10	47/1	9.032	0.003^**^
Married/others	8/37	11/37	0.377	0.539
Body mass index (BMI)	22.16 ± 4.20	21.66 ± 4.62	0.295	0.588
PANSS				
Positive symptom subscale	25.29 ± 7.75	25.48 ± 5.08	0.020	0.888
Negative symptom subscale	22.16 ± 8.96	18.02 ± 6.20	6.673	0.011^*^
General psychopathology subscale	46.18 ± 13.35	38.31 ± 6.31	13.467	< 0.001^**^
Total score	93.62 ± 22.86	81.81 ± 12.30	9.791	0.002^**^
CGI total score	5.47 ± 0.84	5.52 ± 0.77	0.105	0.747
HAMD total score	18.67 ± 12.47	12.65 ± 9.13	7.123	0.009^**^

^*^p < 0.05**,** ^**^p < 0.01.

### Comparison of cognitive function in groups and by sex

Sex cognitive differences in the two groups are summarized in Table 2 on the MCCB total scores and all ten indexes. The control group scored higher than the FEDN schizophrenia group in MCCB total score, Symbol coding, Trail Making A, CPT-IP, Spatial span total, Digital sequence, HVLT-R total, BVMT-R total, Mazes (NAB) total (all *p* < 0.001). Statistical significance was not reached in two areas: Category fluency (*p* = 0.136) and MSCEIT (*p* = 0.120). In the FEDN schizophrenia group, women performed better than men in Symbol coding, Digital sequence, and HVLT-R total (*p* < 0.05). However, there was no sex difference in the other cognitive functions. After controlling for age, smoking, and education, these differences remained significant.

**Table 2:** Comparison of neuropsychological tests between normal controls and FEDN schizophrenia (between sex)

Cognitive domains	Cognitive tests	Normal controls		FEDN schizophrenia		Diagnosis *F* (*p*-value)	Sex *F* (*p*-value)	Diagnosis × sex *F* (*p*-value)
		Male(*n* = 74)	Female(*n* = 86)	Male(*n* = 45)	Female(*n* = 48)			
Speed of processing	Category fluency	56.8 ± 13.1	52.0 ± 12.2	52.8 ± 9.2	51.5 ± 9.7	2.241(0.136)	3.977(0.047)	1.346(0.247)
	Symbol coding	57.7 ± 9.4^*^	55.9 ± 11.6	38.4 ± 11.0^*++^	43.5 ± 9.7^++^	132.753(< 0.001)	1.447(0.230)	6.354(0.012)
	Trail Making A	56.4 ± 8.2	53.8 ± 9.6	45.5 ± 6.8^++^	47.3 ± 7.3^++^	64.223(< 0.001)	0.151(0.698)	4.043(0.045)
Attention	CPT-IP	55.2 ± 7.9	53.5 ± 8.6	40.4 ± 9.7^++^	44.3 ± 9.5^++^	108.633(< 0.001)	0.845(0.359)	6.079(0.014)
Working memory	Spatial span total	60.7 ± 11.4	55.1 ± 11.9	43.7 ± 12.1^++^	48.1 ± 13.2^++^	58.608(< 0.001)	0.170(0.681)	10.297(0.002)
	Digital sequence	60.2 ± 10.5	54.4 ± 10.5	46.0 ± 10.2^*++^	50.8 ± 10.1	43.065(< 0.001)	0.138(0.711)	14.873(< 0.001)
Verbal learning	HVLT-R total	59.8 ± 8.8	56.3 ± 10.3	46.7 ± 12.5^*++^	52.3 ± 9.3^+^	41.215(< 0.001)	0.654(0.419)	11.823(0.001)
Visual learning	BVMT-R total	57.8 ± 8.2^*^	54.8 ± 10.4	49.1 ± 9.5^++^	46.1 ± 10.0^++^	49.165(< 0.001)	5.762(0.017)	0.000(0.994)
Reasoning and problem solving	Mazes (NAB) total	62.4 ± 7.7^**^	56.4 ± 11.0	48.9 ± 10.3^++^	46.6 ± 9.6^++^	83.328(< 0.001)	10.701(0.001)	2.130(0.146)
Social cognition	MSCEIT	51.8 ± 8.8	51.0 ± 8.3	47.7 ± 11.9^+^	51.2 ± 11.1	2.432(0.120)	1.112(0.293)	2.740(0.099)
	Total MCCB scores	63.0 ± 10.6	57.2 ± 12.4	44.2 ± 10.0^++^	47.6 ± 9.7^++^	97.977(< 0.001)	0.665(0.416)	10.234(0.002)

^*^Indicates the comparison between males and females in FEDN schizophrenia or in the controls: ^*^p < 0.05, ^**^p < 0.01

^+^Indicates the comparison between FEDN schizophrenia and the controls in males or females: ^+^*p* < 0.05, ^++^*p* < 0.01

Moreover, multivariate analysis of covariance showed a diagnosis × sex interaction effect for all cognitive domains. To break down the two-way interaction, we examined patients and controls grouped by sex separately. Sex differences were found in Category fluency, BVMT-R total, and Mazes (NAB) total score. The MCCB 6 indexes and total scores showed diagnosis-by-sex interactions, including Symbol coding, Trail Making A, CPT-IP, Spatial span total, Digital sequence and HVLT-R total scores (all *p* < 0.05). However, the significant differences in Symbol coding, Trail Making A, and CPT-IP did not pass the Bonferroni correction (Bonferroni corrected *p* < 0.05/11 = 0.0045).

### Correlation between cognitive function and clinical phenotypes in FEDN schizophrenia patients

Table 3 shows the relationships between multiple clinical characteristics and cognitive deficits, separated by sex. In male patients, Pearson correlation analysis showed significant positive associations between education and multiple cognitive variables. Furthermore, Trail Making A, CPT-IP, Spatial span total, and Mazes (NAB) total score were significantly and negatively associated with the Positive symptom subscale. Only MSCEIT had a negative association with the Negative symptom subscale. Category fluency, Trail Making A, CPT-IP, Mazes (NAB) total score were associated with the General psychopathology subscale. PANSS total score displayed a significantly negative relationship to Trail Making A, CPT-IP, Mazes (NAB) total score, and MCCB total score. The MCCB total score had a significant association with education and PANSS total score. Further multivariate regression analyses showed that education was independently associated with the MCCB total score (beta = 0.407, *t* = 2.726, *p* = 0.010).

**Table 3:** Correlation between MCCB and PANSS and clinical variables in patients with FEDN schizophrenia

	Category fluency	Symbol coding	Trail Making A	CPT-IP	Spatial span total	Digital sequence	HVLT-R total	BVMT-R total	Mazes (NAB) total	MSCEIT	MCCB Total
Correlation with MCCB score (*r*)											
Male (*n* = 45)											
Education	0.471^**^	0.093	0.109	0.322^*^	0.177	0.164	0.213	0.206	0.343^*^	0.194	0.372^*^
Smoking	− 0.303^*^	0.124	0.093	− 0.027	0.039	0.058	− 0.473^**^	− 0.305^*^	− 0.085	− 0.266	− 0.274
BMI	0.101	− 0.013	− 0.006	0.069	0.030	0.062	0.083	0.158	− 0.058	0.038	0.083
Positive symptom subscale	0.280	− 0.042	− 0.341^*^	− 0.360^*^	− 0.366^*^	− 0.140	− 0.243	0.140	− 0.310^*^	− 0.130	− 0.259
Negative symptom subscale	− 0.075	− 0.121	− 0.120	− 0.151	0.134	− 0.133	− 0.170	− 0.121	− 0.080	− 0.309^*^	− 0.215
General psychopathology subscale	0.356^*^	− 0.164	− 0.453^**^	− 0.400^**^	− 0.210	− 0.292	− 0.114	0.029	− 0.301^*^	− 0.038	− 0.255
PANSS total	0.274	− 0.157	− 0.427^**^	− 0.415^**^	− 0.194	− 0.270	− 0.216	0.017	− 0.312^*^	− 0.187	− 0.321^*^
CGI total	0.326^*^	− 0.074	− 0.451^**^	− 0.299^*^	− 0.239	− 0.089	− 0.287	− 0.136	− 0.257	0.062	− 0.254
HAMD total	0.206	− 0.044	0.009	− 0.279	− 0.203	− 0.214	− 0.038	0.088	− 0.147	0.098	− 0.088
Female (*n* = 48)											
Education	0.332^*^	0.469^**^	0.305^*^	0.350^*^	0.513^**^	0.367^*^	0.298^*^	0.399^**^	0.386^*^	0.315^*^	0.535^**^
Smoking	− 0.047	− 0.211	− 0.248	− 0.158	− 0.122	− 0.084	− 0.164	− 0.158	− 0.227	− 0.079	− 0.221
BMI	0.126	0.199	− 0.013	0.119	0.155	0.033	− 0.082	0.032	− 0.103	0.129	0.068
Positive symptom subscale	− 0.075	− 0.030	− 0.022	− 0.118	− 0.012	− 0.144	− 0.123	0.009	0.171	− 0.145	− 0.069
Negative symptom subscale	− 0.339^*^	− 0.279	− 0.244	− 0.321^*^	− 0.308^*^	− 0.284	− 0.470^**^	− 0.279	− 0.196	− 0.460^**^	− 0.475^**^
General psychopathology subscale	− 0.143	− 0.242	− 0.327^*^	− 0.258	− 0.105	− 0.244	− 0.407^**^	− 0.173	− 0.114	− 0.335^*^	− 0.339^*^
PANSS total	− 0.276	− 0.277	− 0.300^*^	− 0.343^*^	− 0.214	− 0.328^*^	− 0.497^**^	− 0.225	− 0.087	− 0.463^**^	− 0.442^**^
CGI total	− 0.342^*^	− 0.046	− 0.084	− 0.101	− 0.191	− 0.214	− 0.227	− 0.051	− 0.056	− 0.480^**^	− 0.276
HAMD total	− 0.008	0.053	− 0.198	0.179	0.102	0.382^**^	0.057	0.048	− 0.003	− 0.067	0.101

^*^p < 0.05**,** ^**^p < 0.01

In female patients, Pearson correlation showed significant positive correlations between education and MCCB ten indexes and MCCB total score (all *p* < 0.05). Except for Symbol coding, Trail Making A, Digital sequence, BVMT-R total, and Mazes (NAB) total score, the other cognitive domains were negatively associated with the Negative symptom subscale. Trail Making A, HVLT-R total, MSCEIT, and MCCB total score were negatively associated with the General psychopathology subscale. PANSS total score was significantly negatively related to Tail Making A, CPT-IP, Digital sequence, HVLT-R total, MSCEIT, and MCCB total score. Finally, we found an association between MCCB total score and education, PANSS negative symptom and general psychopathology subscale scores, and PANSS total score. Further multivariate regression analyses showed that the following variables were independently associated with the MCCB total score: education (beta = 0.425, *t* = 3.730, *p* = 0.001), the PANSS Negative symptom subscale (beta = − 0.308, *t* = − 2.561, *p* = 0.014), the PANSS General psychopathology subscale (beta = − 0.319, *t* = − 2.145, *p* = 0.038), HAMD total score (beta = − 0.299, *t* = − 2.422, *p* = 0.020).

## Discussion

To the best of our knowledge, this is the first study of sex differences in cognitive impairment with first-episode drug-naïve schizophrenia in China. The main finding of our current study is that patients with schizophrenia have demonstrable cognitive dysfunction. Additionally, there were apparent sex differences in cognitive impairment with FEDN schizophrenia in this sample. Male patients performed worse than female patients in symbol coding, digital sequence, and verbal learning. Interestingly, we also found six indexes and MCCB total score that showed diagnosis-by-sex interactions, belonging to the speed of processing, attention, working memory, and verbal learning. Lastly, sex differences in cognitive impairment were significantly related to multiple clinical symptoms and general characteristics (Table 3).

A great deal of research and analysis has been devoted to evaluating the neuropsychologic disorders suffered by schizophrenia patients in several cognitive fields. Language ability, executive function, attention, and the ability to filter irrelevant stimuli are all impaired. Working memory and executive function of patients with schizophrenia are also impaired [27–29]. Consistent with this finding, several cognitive deficits have been reported in adolescent schizophrenia. For example, Victoria et al. used MCCB to examine cognitive impairment samples in Mexican adolescents with schizophrenia. After 3 and 6 months of treatment, all domains were improved except for social cognition [30]. In recent years, oxytocin (OXT) has emerged as a novel strategy for treating social cognitive and social behavioral deficits in schizophrenia-spectrum disorders, an intriguing prospect from both the evolutionary perspective and the neurodevelopmental-cognitive model. Therefore more research is needed to determine the utility of OXT as a treatment option or adjuvant therapy for schizophrenia [31, 32]. Other preliminary studies have shown that male patients with chronic schizophrenia have more severe cognitive impairment than female patients in immediate memory and delayed memory. However, these differences were not found in language, visuospatial or attention indices [33]. The difference between schizophrenia and normal control is the result of a combination of factors. An in-depth study of these differences can help to guide the treatment in the future.

Stress has been shown to damage memory that leads to cognitive impairment in multiple clinical contexts. Corticotropin-releasing factor (CRF) likely plays a primary role in mediating stress mnemonic dysfunction. Wiersielis assessed whether the projection of CRF into the medial septum (MS) of the hippocampus would affect memory formation in male and female rats [34]. Interestingly, the results indicated that males are more vulnerable than females to be affected by the memory impairment caused by CRF in the MS. This may explain why schizophrenia is more common in men and why men usually show more significant cognitive impairment. In men and women, CRF1 antagonists can prevent MS-mediated memory impairment caused by high CRF levels, which may be related to stressful events. Collectively, CRF1 antagonists may be a viable option for treating cognitive impairment in stressed individuals with mental disorders.

Another possible reason for the demonstrated sex differences in the clinical presentation of schizophrenia may be the biological differences in sex hormones. Women often display more mild symptoms, and one hypothesis is that estrogen may have a protective effect on schizophrenia. The relationship between estrogen and BDNF, NMDA receptors, GABA receptors, and the luteinizing hormone may be an essential way to understand sex differences [35]. Estrogen has therapeutic effects and exerts neuroprotective effects, including anti-excitotoxicity and oxidation. Another prominent female gonadal hormone is progesterone, and available data indicate a critical modulator in regulating the central system through the dopaminergic system [36].

Although women and men with schizophrenia show similar neuropsychological damage [37]**,** the available evidence strongly supports sex differences in neuropsychological performance. Female patients have a later age of onset, better functional outcomes, less adverse symptomatology, cognitive impairment, and more severe positive symptoms [38]. Nevertheless, consistent with the studies mentioned above, our study showed that male patients with schizophrenia performed worse in symbol coding, digital sequence, and verbal learning in the first-episode schizophrenia group. However, there were no gender differences in other cognitive functions. We also found that men are significantly worse than women on PANSS total score, Negative symptom scale, General psychopathology scale, and HAMD total score. Consistent with this finding, Li found sex differences in first-episode psychosis from 360 patients in Hong Kong participants diagnosed with mental illness for the first time between the ages of 26 and 55. They had received antipsychotic treatment for less than 12 months. In women, memory was significantly associated with onset age, negative symptoms, and side effects. Selective attention was correlated with the age of onset and education in men and positive symptoms and short-term symptoms [12].

For sex differences in cognitive deficits, Zhang enrolled 248 patients with chronic schizophrenia and 188 healthy controls. Using the Repeatable Battery for the Assessment of Neuropsychological Status (RBANS), the brain-derived neurotrophic factor (BDNF) levels were lower in patients with chronic schizophrenia. Furthermore, male patients with schizophrenia had significantly lower BDNF and more unsatisfactory memory performance than their female counterparts, and in female patients, BDNF correlated significantly with immediate and delayed memory. There was no gender difference in the normal control group [38]. In addition, the impairment of visual perceptual organization ability is a cognitive defect repeatedly observed in patients with schizophrenia, but we did not find differences in visual learning between genders. There are inconsistencies in the literature on sex differences in these cognitive deficits. A Spanish study enrolled 74 female and 86 male participants who suffered from the first episode of psychosis. Although women scored higher than men on verbal memory, men scored higher than women on reaction time, visual memory, and planned tasks. In that study, there were no gender–group interactions in any of the neuropsychological tests [14].

There are some limitations to this study. First, this cross-sectional design cannot demonstrate the longitudinal course of illness that long-term research might. Second, the patients' age in the first-episode drug-naïve schizophrenia group was younger than that of the control group. The inclusion criteria of symptoms less than 60 months and the antipsychotic naivete likely skewed the age younger in this group. Nevertheless, the effect of these data on cognitive function is more useful and less confounded than in patients who have received long-term treatment for schizophrenia. Third, although we initially enrolled more patients, the sample size diminished due to exclusion criteria, incorrect questionnaires, and incomplete cognitive assessment. Finally, we chose MCCB as the cognitive testing, which may have data bias. More measurements and laboratory data need to be collected to evaluate better cognitive impairment and sex differences in patients with schizophrenia. In the future, the sample size should ideally be expanded, characteristics such as education controlled for, and longitudinal studies should be conducted to track cognitive changes.

## Conclusions

Our results suggest that there is cognitive dysfunction in the schizophrenia group. Specifically, men with FEDN schizophrenia have lower cognitive abilities than women in symbol coding, digital sequence, and verbal learning. Future studies should also consider the possible causes of sex differences in patients with schizophrenia, and appropriate strategies should be implemented, especially in evaluating the influence of treatment and longitudinal course of schizophrenia.

## Data Availability

The datasets used and/or analyzed during the current study are available from the corresponding author on reasonable request.
